# Reversal learning is influenced by cognitive flexibility and develops throughout early adolescence

**DOI:** 10.1038/s41539-025-00308-3

**Published:** 2025-05-12

**Authors:** Christoph Bamberg, Sarah Weigelt, Klara Hagelweide

**Affiliations:** 1 Department of Vision, Visual Impairment and Blindness Technical University Dortmund, Dortmund, Germany; 2 Department of Psychology Paris Lodron University of Salzburg, Salzburg, Austria

**Keywords:** Human behaviour, Human behaviour

## Abstract

Learning behavioural responses and adapting them based on feedback is crucial from a young age, continuing to develop into young adulthood. This study examines the development trajectory and contributing factors from childhood to adulthood using a reversal learning paradigm. We tested 202 participants aged 10 to 22 in an online study, where they learned and reversed stimulus-outcome associations in a new blocked design paradigm and were assessed for working memory capacity. Results showed that reversal learning performance improved with age, particularly for 10- to 14-year-olds. Flexible responses to negative feedback correlated with better reversal learning. Additionally, pubertal development and working memory were positively associated with reversal learning. These findings align with previous research, highlighting flexible feedback responses as a key factor in reversal learning. As the overall rate of flexible reactions did not change with age, it could support reversal learning independent of age, potentially changing its role during development.

## Introduction

Learning is a life-long process. Not only what we learn is the result of a continuous process of adaptation, the very way we learn changes through our lifetime (e.g.^1,2^). For instance, adults have a staggeringly different approach to learning a new language than infants, who begin to speak without even having a concept of language, indicating that learning does not just gradually increase in quantity, but also changes its quality during development^3^.

In adolescence in particular, both improvements and qualitative changes in learning processes are accompanied by far-reaching restructuring of the brain^4,5^. In this way, accelerated growth and maturation go together with an expansion of cognitive, affective, and self-regulatory abilities that pave the way for the transition from child- to adulthood^6^. Neuroscientific studies show that changes in the brain that begin at puberty alter social information processing^7^ and the intake, integration, and retrieval of information in general^8,9^. Accordingly, developments in underlying reinforcement learning processes are assumed to shape social behaviour and social learning during adolescence^10,11^. Notably, adolescents do not automatically outperform children in all learning tasks or situations. For instance, fear extinction, i.e., the unlearning of a conditioned fear response, seems to be slowed in adolescents compared to both children and adults^12,13^. This observation is supported by studies in juvenile rodents^14^ and can be related to developmental plasticity in the neuronal circuits underlying fear conditioning^2^. Furthermore, children seem to be more receptive to statistical properties of phonetic input^15^ and motor sequences^16^ than adolescents and adults and were found to be more open to unusual causal relations than older participants^1^. These studies show the complexity of learning and point to the importance of investigating the developmental trajectories of different learning processes and their underlying mechanisms. Research on one aspect of learning, namely incorporating instrumental feedback, shows significant changes in adolescence that are yet not fully understood.

Reinforcement learning includes the ability to form stimulus-outcome associations via instrumental feedback. As a simple example of instrumental reinforcement learning tasks, participants may get positive feedback if they press a button in reaction to a blue triangle, but negative feedback if they press the same button in reaction to a red square. This feedback is instrumental in that it informs the individual on how to adapt their response and increase the associated reward of their response. Oftentimes feedback is provided in a probabilistic way, so that, for example, in 80% of trials the blue triangle is associated with “correct” feedback and in 20% with “incorrect”, making it more challenging to learn the dominant association^17^. The ability to learn associations from probabilistic information was found to increase with age, especially during adolescence^18–20^. Nevertheless, in some studies no age-dependent performance differences were observed^21^, or adolescents performed superior to adults when false conceptual information misguided the older participants^22^ or even in a simple probabilistic learning task^23^. Hence, while instrumental learning seems to generally improve with age^11^, specific aspects of it may follow a more complex trajectory^24^.

In which way learned associations are adapted to new circumstances is one important aspect that may help understanding the intricate development of instrumental learning. Reversal learning (RL) paradigms test the ability to revise stimulus-outcome associations by changing instrumental feedback after participants learned to exhibit a certain response to a stimulus. Following the example from above, a button press in reaction to the blue triangle may suddenly turn out to be “incorrect”, reversing its association. Research generally indicates that RL ability increases during adolescence^25–29^. This might be based on an increase of brain signal variability between childhood and mid-adulthood^30,31^. Nevertheless, results are not consistent. For instance, Eckstein and colleagues found the most optimal learning strategies and highest RL performance in adolescents (13–15 years) compared to both children and adults^32^. Similarly, in a more recent study by Eckstein and colleagues of 291 participants aged 8–30 using a reversal-learning task designed to test the balance of persistence and flexibility, adolescents in their mid-teen years were found to outperform both younger and older age groups^30^. A possible peak of RL in adolescence is supported by some evidence from rodent studies: For instance, juvenile mice performed better on an odour discrimination task with reversals than adult mice^33^. On the contrary, Hauser and colleagues^34^ did not observe performance differences of adolescents (12 - 16 years old) and adults in their RL paradigm. Nevertheless, using a computational modelling approach for the employed learning strategy, they found that adolescents learned faster from negative reward prediction error than adults, i.e., they showed higher sensitivity to changes in reward expectations. Similarly, van den Bos and colleagues did not find behavioural differences in learning associative rules comparing 8- to 11-year-olds, 13- to 16-year-olds and 18- to 22-year-olds in a probabilistic associative learning task. Nonetheless, they did find differences in neural activation between children and adults when comparing the processing of positive vs. negative feedback. These studies highlight that changes in the processing of negative feedback should be considered when investigating the development of RL performance during adolescence.

Indeed, studies found that children, adolescents, and adults use negative feedback to a different extent to change their behaviour: A study comparing 8- to 9-year-olds with 11- to 13-year-olds and 18- to 25-year-olds indicates that only children learn less well from negative instrumental feedback i.e., the information that a former response was incorrect, compared to positive instrumental feedback^21^. Van der Schaaf et al. found that especially adolescents between 16 and 17 years learn most strongly from negative instrumental feedback, compared to both children and adults^27^. Reviewing neurodevelopmental research, DePasque and Galván assume that the prefrontal control to learn from negative feedback might develop during adolescence and that its effect on instrumental and reversal learning depends on task properties such as the informative value and quality of feedback^18^. They further suggest that adolescents exhibit greater cognitive flexibility in response to negative feedback compared to adults, which might lead to an advantage especially seen in reversal learning tasks^29^. Moreover, this may be driven by increased dopamine-driven flexible behaviour in adolescence^35^.

To summarise this body of research, the ability to reverse learned stimulus-outcome associations seems to increase from childhood to adulthood. Rapid changes in performance during adolescence and sometimes superior performance compared to adults were observed, although not always on the level of reversal performance but in relation to the underlying handling of instrumental feedback. This suggests that not only RL changes with age, but the way feedback is used to form new stimulus-outcome associations is subject to developmental change as well. Only a few studies investigated to what extent and in what way children and adolescents use negative instrumental feedback during RL compared to adults, although it seems to be an important factor contributing to the idiosyncratic RL performance in adolescence. To better understand these two aspects - the developmental trajectory of RL and the importance of reacting to negative feedback for RL performance - we designed a novel RL paradigm.

The reviewed studies on the development of RL mostly used a “continuous” design, i.e., stimulus-outcome associations reversed after a fixed number of correct answers learned from instrumental feedback^25,34,36^. Here, we take a different approach with a new paradigm by guiding the participant’s learning throughout the course of the experiment. The paradigm is based on an associative learning task by Kinner and colleagues (ref. ^37^; adapted from^38^) that was modified to provide probabilistic feedback (80% valid and 20% invalid feedback trials). The paradigm uses a blocked design where invalid feedback appears at the beginning and end of each block, with valid trials in the middle. True stimulus-outcome associations may change between blocks, requiring participants to determine whether feedback signals a genuine change or is misleading. A key focus is how flexibly participants adapt their responses to negative feedback. Furthermore, participants can properly learn the associations before a reversal takes place, as at least eight consecutive valid trials are provided in the middle section of a learning block for each stimulus. Previous paradigms mostly used shorter phases of valid feedback (e.g., five trials) and more reversals. On the one hand, this set-up makes it more feasible to control the difficulty of the paradigm, so that participants at the age of 10 years manage to learn the associations even via probabilistic feedback. On the other hand, this helps distinguishing the associative-learning aspects from those related to flexibly reacting to feedback: We can measure the participants’ tendency to react flexibly to negative instrumental feedback (henceforth referred to as *flexible reactions*, omitting the qualification “to negative instrumental feedback”) at the beginning of each of three learning blocks independent of overall RL performance. Furthermore, we can observe the participants’ ability to adapt their response strategy according to the overall structure of the experiment: Reacting flexibly in the first learning block can be interpreted as openness to meaningful feedback; this is also true in the second and third block, as a break between learning blocks and half of the stimuli occurring in a new context indicate a potential change in contingency. Nevertheless, a decrease in flexible responses over the three blocks indicates an adaptation to the paradigm’s structure: Since feedback at the beginning of a block is partly misleading, it is most effective to stick to the association learned in the previous block and change the association only after repeated negative feedback.

We also added several questionnaires to the study to identify other age-dependent factors that may influence task performance next to the tendency for flexible reactions. Studies indicate that higher working memory has a positive impact on cognitive tasks in general (e.g.^39–42^, and is related to better probabilistic learning performance^43^. As it has not been clarified yet if this relationship changes from child- to adulthood^19^, we included a measure of working memory capacity to test its relation to reversal learning performance. Furthermore, we collected the motivation to engage in an effortful cognitive task, measured by the need for cognition^44^, as a possible factor that might interact with the effect of working memory capacity on performance, especially during adolescence^45,46^.

In sum, in this study we used a new paradigm to investigate developmental changes in RL performance, its relation to working memory and need for cognition, and the role of a specific aspect of the learning process, the reaction to negative instrumental feedback.

The main questions of interest in this study are how RL performance develops from early adolescence to young adulthood and how it is related to flexible reactions. We also ask how these flexible reactions differ between age groups, i.e., between early-adolescents (10 to 13 years), mid-adolescents (14 to 17 years) and young adults (18 to 22 years). Furthermore, we explore the relation of working memory capacity and the motivation to engage in effortful cognitive processes to RL performance. We preregistered further hypotheses focusing on probabilistic learning, the role of valence and contextual information on performance, and the stability of responses. These questions are beyond the scope of this article and will be considered in a later publication (see the public preregistration on the OSF repository: https://osf.io/ep5ua).

The hypotheses that we investigate here are the following: In line with the majority of findings on RL during development, we expect older participants to learn reversed stimulus-outcome associations better (H1a) and faster than younger participants (H1b). According to the studies on the role of negative feedback, this RL performance is hypothesised to be generally higher in participants that react flexibly to negative instrumental feedback (H2a). Nevertheless, we expect that the mid-adolescents’ openness to a behavioural change after negative feedback may override the learned prediction of the correct response, hindering RL instead of facilitating it. Therefore, the association between RL performance and flexible responses is expected to be positive in adults and early-adolescents, but non-existent or negative in mid-adolescents (H2b). Based on a study that investigated reactions to negative instrumental feedback in adolescents^27^ we expect that flexible reactions are higher in mid-adolescents compared to early-adolescents and adults, following an inverted-U trajectory (H3a). Additionally, we hypothesised adults to optimise their response strategy more successfully than adolescents, i.e., adults should decrease their flexible reactions throughout the experiment as a sign of strategy adaptation, while early- and mid-adolescents should not (H3b). Finally, going further into the factors underlying successful RL, we expected working memory capacity and the motivation to engage in an effortful cognitive process to be both positively associated with RL performance (H4a), that motivation is more crucial for younger than older participants, so that the effect of motivation decreases with age (H4b), and working memory and motivation to interact in their effect on RL (H4c). In addition, we explored whether there are sex differences for these outcomes.

## Results

In our analysis, we first considered the necessary precursor for reversal learning, namely that the initial stimulus-outcome associations are learned, before they can be reversed. After confirming this, we examined the relation between age and RL (H1a, H1b) as well as the association between puberty scores and reversal learning. Third, we investigated the link between flexible reactions and RL (H2a). Fourth, we assessed age group differences in these flexible reactions and their change during the experiment (H2b, H3a, H3b). Lastly, we explored the effects of motivation and cognitive capacity on performance in our paradigm (H4a-c). Here, we report the statistics without outliers removed. However, the supplementary analysis includes corrections for data points that yield a strong influence on the regression, which were excluded if their Cook’s D was above one. This additional caution did have only a minor impact on the results.

### Did Participants Learn the Initial Associations?

RL presupposes proper learning of a stimulus-outcome association. Hence, we excluded participants who did not perform above our pre-specified initial learning threshold in the first learning block (*N* = 54, 27%, see Supplementary Table 1). They were especially younger participants (logistic regression for above/below threshold: *coeff age* = −0.15, SE = 0.04, 95% CI = [−0.2, −0.03], *p* = 0.006). After excluding these participants, the sample analysed below is *N* = 142; mean age *M* = 17.32, *SD* = 3.74 (see Table 1) of whom 32% are male (see Supplementary Table 2).

**Table 1:** Age Distribution Of The Final Sample

Age	10	11	12	13	14	15	16	17	18	19	20	21	22	Total
N	4	13	8	12	8	9	12	10	8	10	12	22	14	142

As a next step, we considered how well participants learned stimulus-outcome associations over the whole experiment. The pattern of learning performance in the different age groups is as expected: Early-adolescents performed significantly worse in the second and third block compared to the first learning block (see Supplementary Table 2.; Supplementary fig. 1). In contrast, mid-adolescents’ performance is only significantly decreased for the third compared to the first learning block. The performance of adults did not change significantly from learning block one to two or three.

This shows that the paradigm allowed learning the stimulus-outcome associations and elicited differences between age groups as planned. After this initial confirmation of our approach, we turn to our hypotheses on RL.

### Does Reversal Learning Differ Between Age Groups?

**Fig. 1:**
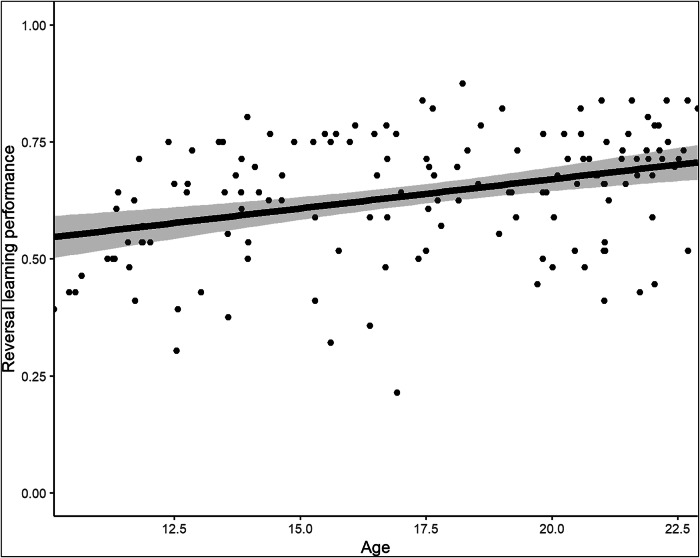
Scatterplot For Age and RL Performance. The solid line is the estimated coefficient. The shaded area shows the 95% Confidence Interval. The plot shows the participants’ age on the x-axis and their RL performance on the y-axis.

**Fig. 2:**
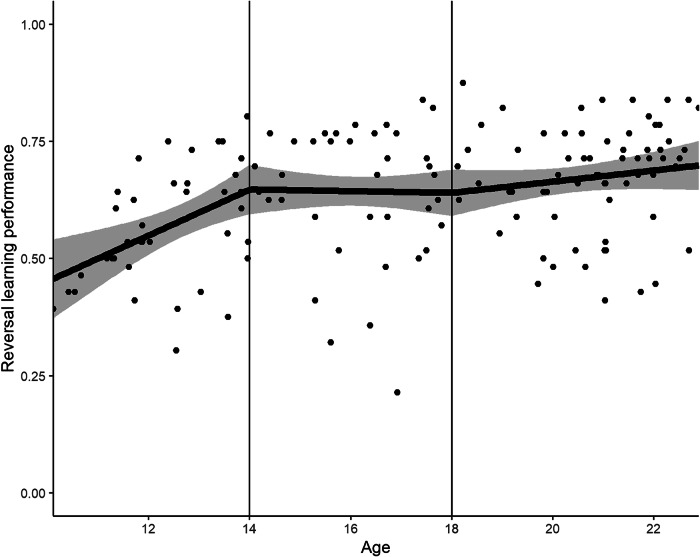
Linear Basis-Spline Regression for Age and RL Performance. The segmented regression line (solid line) with 95% Confidence Intervals (shaded area) and the knots at 14 and 18 years are shown. Age is plotted on the x-axis and RL performance on the y-axis.

We observed the expected age-dependent increase in RL performance: For an increase in age by one year, the estimated improvement in RL performance is 0.01 units (SE = 0.003, 95% CI = [0.01, 0.02], p < 0.001; Fig. 1). This means that, for example, a 17-year-old participant compared to a 10-year-old participant is estimated to have a reversal score 0.07 units higher on a scale from zero to one. This association remained when adding sex as a covariate (see below). To better understand this relation between age and RL, we fitted a regression spline model with separate slopes for early-, mid-adolescents and adults (Fig. 2). For early-adolescents (between the y-intercept and the first knot at 14 years), the estimated slope is 0.05 (see Supplementary Table 3); for mid-adolescence it is −0.002; and for adults 0.01. This indicates that the relationship between age and RL performance is strongest in early-adolescents between 10 and 14 years compared to the rest of the sampled age range.

As a next step, we investigated whether this relation is also true for RL response times (H1b). Against our expectations, we did not find a significant linear decrease in log-response times with age (coeff = −0.01; SE = 0.01, 95% CI = [−0.03, 0.001], *p* = 0.086). Yet, when excluding response times below 200 ms., since they are too fast to be serious responses to the task, the relation with age is significant (coeff = −0.02; SE = 0.01, 95% CI = [−0.03, −0.001], *p* = 0.011).

### Does Reversal Learning Differ Depending on Pubertal Maturation and Sex?

As an exploratory step, we assessed how much of the developmental change in RL performance can be explained by self-reported puberty levels for underage participants (*N* = 75, pubertal development data for one participant was missing). With every unit increase in puberty, the RL performance increases by 0.2 units (SE = 0.09, 95% *CI* = [0.008, 0.38], *p* = 0.04).

There were approximately twice as many female than male participants in the sample (see Supplementary Table 5). Sex as well as age show a significant association with RL performance when adding them to the same regression model (*coeff female sex* = 0.05; SE = 0.02, 95% CI = [0.001, 0.09], *p* = 0.045; *coeff age* = 0.01; SE = 0.003, 95% CI = [0.01, 0.02], *p* < .001).

In sum, RL performance was slightly higher for older participants, especially within the early-adolescent group. Furthermore, it increased with pubertal development and was higher with female sex but did not co-occur with faster response times.

### What is the Relation between Reversal Learning Performance and Flexible Reactions to Negative Instrumental Feedback?

**Fig. 3:**
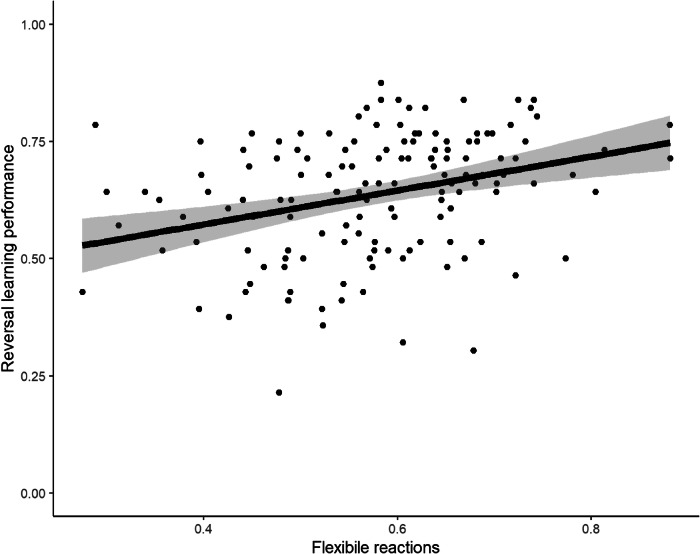
Scatterplot For Flexibility and Reversal Learning Performance. Flexible reaction scores are plotted on the x-axis, RL response times on the y-axis. The solid line is the estimated coefficient. The shaded area shows the 95% confidence interval.

After establishing a general relation between maturation and RL performance, we turned in more detail to a specific determinant of RL: flexibly reacting to negative instrumental feedback. As hypothesised (H2a), there is a positive association between RL performance and flexible reactions: For a 0.1 increase in flexible reactions (scale from 0 to 1), there is a 0.036 increase in reversal learning performance (SE = 0.09, 95% CI = [0.18,0.54], *p* < 0.001; Fig. 3). This effect remained significant when controlling for sex as a covariate (see Supplementary Table 6).

### How Do Age and Flexible Reactions Together Contribute to Reversal Learning?

We found age and flexible reactions to be associated with RL performance in independent regression models. When using them both in the same regression model, i.e. controlling for the variance explained by the other factor, each of them still significantly explains RL performance (coeff age = 0.01, SE = 0.003, 95% CI = [0.006, 0.02], *p* < 0.001; coeff flexible reactions = 0.32, SE = 0.09, 95% CI = [0.14, 0.49], *p* < 0.001). This indicates that both higher age and a stronger tendency to change one’s answer after negative feedback contribute to better RL performance.

**Fig. 4:**
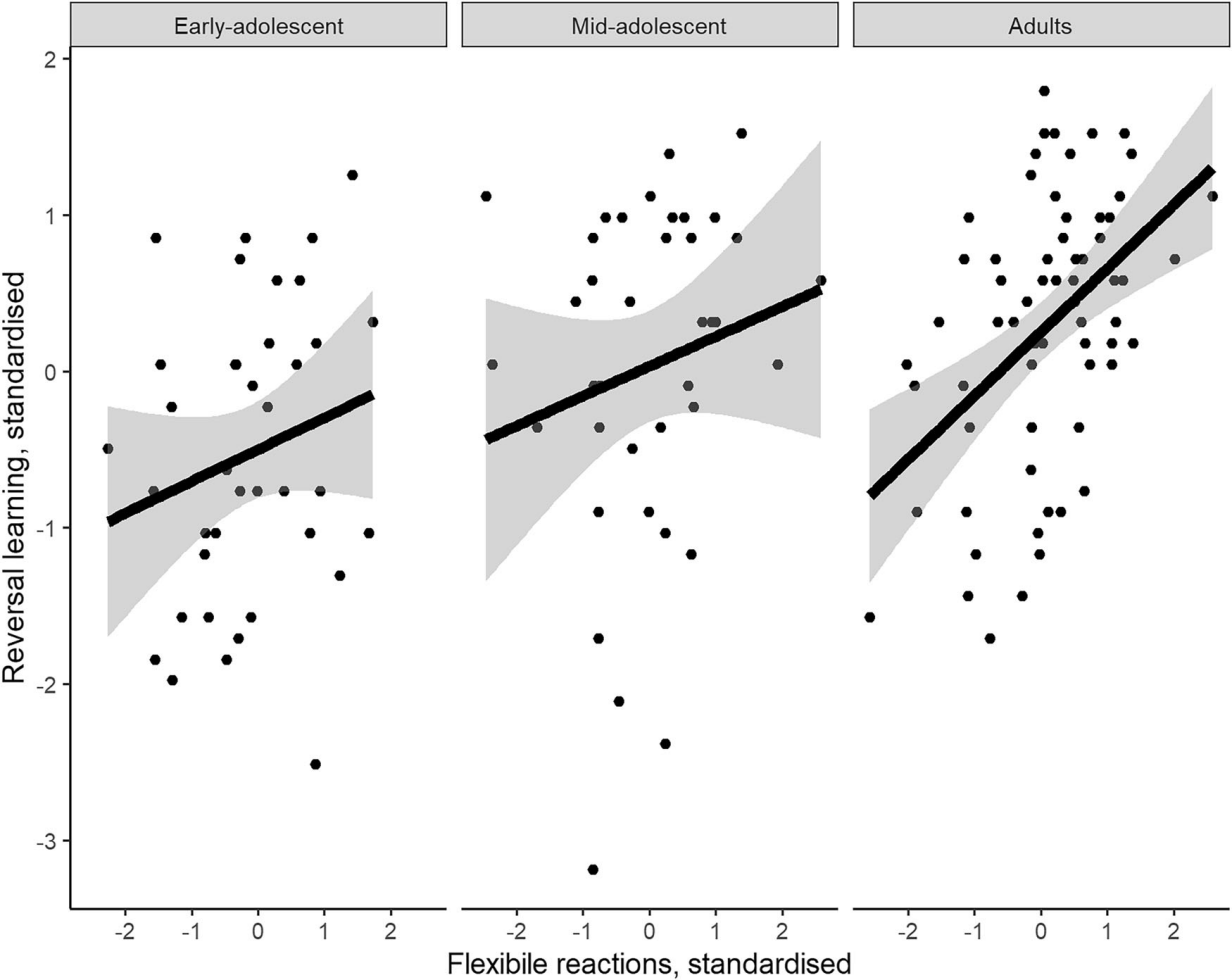
Relationship Between Flexibility and RL Performance for Early-Adolescents, Mid-Adolescents and Adults. The plot shows separate regressions for each age group. Variables are standardised.

Moreover, we hypothesised both factors to interact in a specific way (H2b): better performance in learning reversed contingencies should be associated with more flexible reactions in adults and early-adolescents while we expected this association to be non-existent or negative for mid-adolescents, based on previous research. To test this hypothesis, we fitted linear regressions with the predictor flexible reactions separately for early-adolescents, mid-adolescents, and adults. Indeed, results show no significant relation between flexible reactions and RL performance in early-adolescents or mid-adolescents while a significant association was observed in adults (Table 2; Fig. 4).

**Table 2:** Results of Regressions with Flexible Reactions for Each Age Group

Age group	Variable	Coefficient	SE	95%CI	*P*
Early adolescent	Intercept	0.44	0.10	[0.23, 0.64]	< 0.001
	Flexible reactions	0.23	0.18	[−0.13, 0.59]	0.201
Mid adolescent	Intercept	0.52	0.11	[0.29, 0.74]	< 0.001
	Flexible reactions	0.22	0.19	[−0.17, 0.60]	0.263
Adult	Intercept	0.40	0.07	[0.27, 0.54]	< 0.001
	Flexible reactions	0.46	0.11	[0.24, 0.68]	< 0.001

Nevertheless, when we predicted RL performance by age, flexible responses and the interaction between the two variables in a regression model, therefore assuming a linearly increasing influence of age on the positive effect of flexible reactions on RL, we found no significant interaction between age and flexible reactions (coeff = 0.03, SE = 0.02, 95% CI = [−0.01, 0.07], *p* = 0.174) while age and flexible responses still significantly predicted RL performance. Taken together, these results indicate that participants with a higher tendency for flexible reactions performed better, irrespective of their age, but that his relation was most pronounced for adults.

### What is the Relation Between Age and Flexible Reactions to Negative Feedback?

**Fig. 5:**
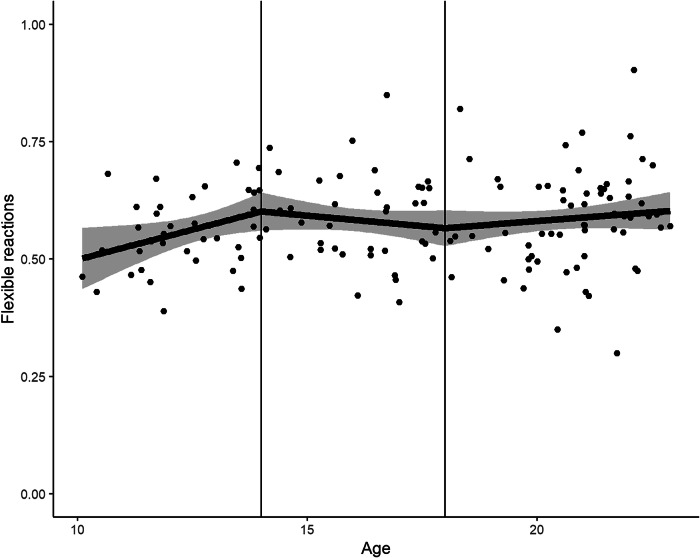
Linear Basis Spline Regression for Age and Flexible Reactions. Age is plotted on the x-axis, flexible reactions on the y-axis. The solid line is the estimated linear basis spline regression. The shaded area shows the 95% confidence interval. The knots at 14 and 18 years are shown as vertical lines.

To test our hypothesis that flexible reactions are highest in mid-adolescence, we fitted a model with a linear and quadratic term for age as a coefficient to flexible reactions as an outcome. We neither found evidence for a quadratic relation between age and flexible reactions (coeff = −0.01, SE = 0.01, 95% CI = [−0.03, 0.01], *p* = 0.56) nor a linear relation (coeff = 0.01, SE = 0.01, 95% CI = [0.0, 0.03], *p* = 0.16), with no change when adding sex as covariate (see Supplementary Table 7). Since a quadratic term for age did not describe the development in flexible reactions well, we conducted an exploratory basis-spline regression for flexible reactions (Fig. 5). We only found significant basis functions for early-adolescents and adults. The slope for early-adolescents, 0.03, was three times as large as for adults, 0.01 (see Supplementary Table 4). The slope for mid-adolescents is −0.01.

**Fig. 6:**
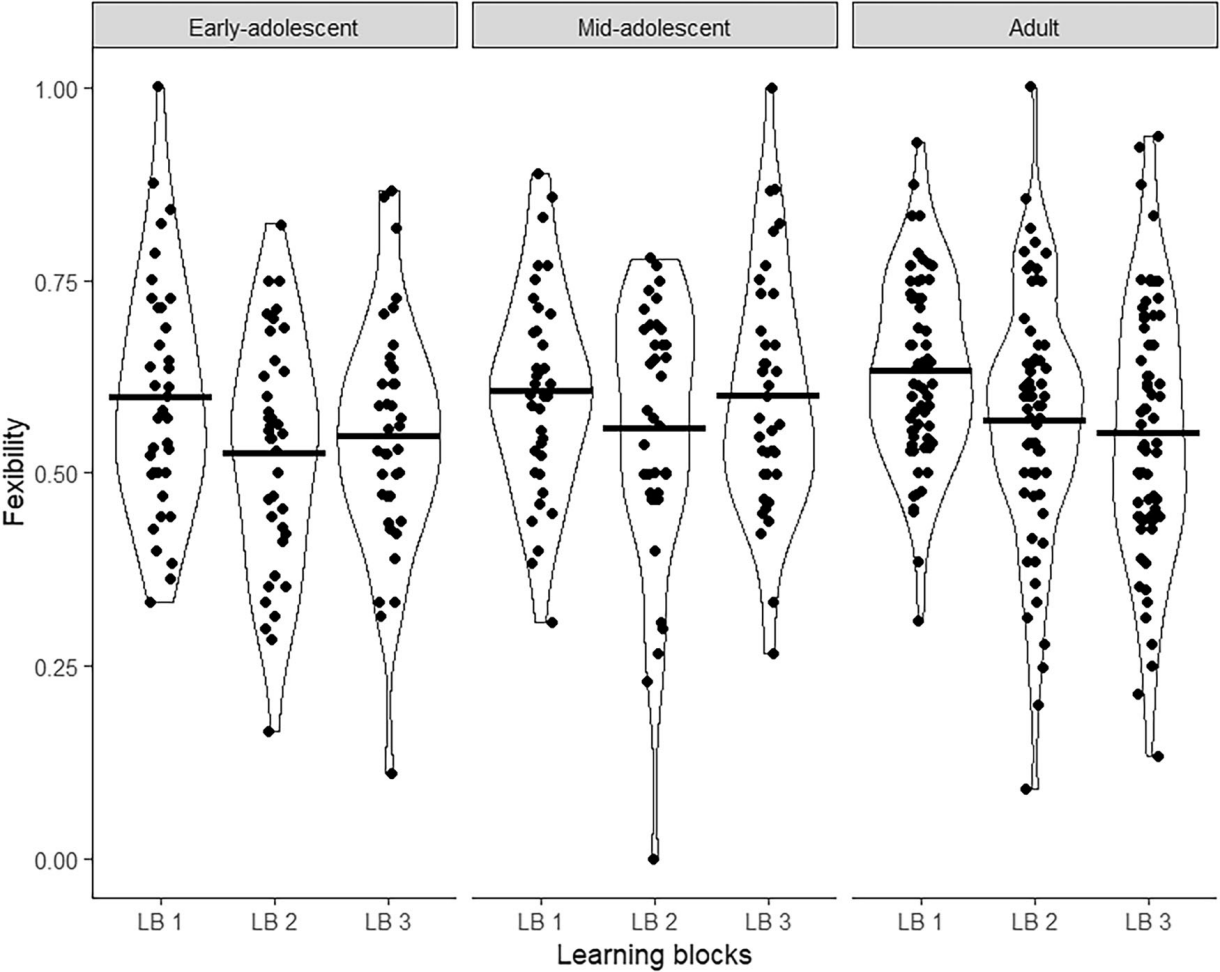
Change In Flexible Reactions Over Learning Blocks, Split By Age Group. Learning block one to three are plotted on the x-axis for early-adolescents, mid-adolescents and adults. Flexible reactions in the respective learning block is plotted on the y-axis. Horizontal bars are means, the density is given by the shape of the violin plot and data points are slightly jittered.

After considering the flexible reactions throughout the whole paradigm, we also investigated differences between the three learning blocks. We anticipated that adults would reduce flexible reactions in response to negative feedback, indicating that they saw through the pattern of invalid feedback in the beginning of a block, while adolescents would not show this adaptation (H3b). To assess this, we applied linear regression models to each age group, using the learning block as a categorical predictor with the first block as the intercept. In early-adolescents, flexibility significantly decreases from first to second learning block Table 3; Fig. 6) but not from first to third learning block. In mid-adolescents, there is neither a significant change in flexibility from learning block one to two nor from one to three. In adults, flexibility significantly decreases between first and second learning block and first and third learning block. Notwithstanding the reduction from first to second learning block in early-adolescence, only adults showed the expected decrease in flexible reactions in both learning block two and three, indicating that they may have understood this feedback in the beginning of the learning blocks to be invalid.

**Table 3:** Flexible Reactions in Learning Blocks, Split by Age Groups

Age group	LB	Coefficient	SE	95% CI	p
Early adolescent	LB 1 (Intercept)	0.60	0.02	[0.55, 0.67]	< 0.001
	LB 2	-0.07	0.03	[−0.14, −0.003]	0.040
	LB 3	-0.05	0.03	[−0.12, 0.02]	0.154
Mid adolescent	LB 1 (Intercept)	0.60	0.03	[0.56, 0.65]	< 0.001
	LB 2	-0.05	0.04	[−0.12, 0.02]	0.181
	LB 3	-0.005	0.04	[−0.07, 0.07]	0.890
Adult	LB 1 (Intercept)	0.63	0.02	[0.60, 0.67]	< 0.001
	LB 2	-0.06	0.03	[−0.12, −0.01]	0.015
	LB 3	-0.08	0.03	[−0.13, −0.03]	0003

### Influences of Working Memory Capacity and Need for Cognition

Besides testing the relation between age, flexible reactions and RL, we also investigated whether there is an association with working memory and Need for Cognition (NFC) as two likely candidates of situational and personality factors, respectively, that may influence RL (H4a-c). We found a positive association between working memory and reversal learning performance (coeff = 0.03, SE = 0.01, 95% CI = [0.009, 0.053], *p* = 0.007). However, NFC and RL performance were not significantly associated (coeff = 0.014, SE = 0.01, 95% CI = [−0.006, 0.04], *p* = 0.18). There was no interaction between working memory or NFC and age in their effect on RL. Interestingly, in an exploratory analysis, working memory was significantly associated with flexibility (coeff = 0.02, SE = 0.004, *p* < 0.001, 95%CI = [0.007, 0.02]). Moreover, working memory was higher for older participants (coeff = 0.08, SE = 0.02, 95% CI = [0.04, 0.13], *p* < .001).

## Discussion

We investigated how RL differs between age groups and in relation to flexible reactions to negative instrumental feedback. Our main finding is that older participants learned stimulus-outcome association reversals slightly better than younger participants, which is consistent with our first hypothesis. This was especially the case for early adolescents and may be accounted for by pubertal development. Reaction times did not change in relation to age according to our preregistered analysis. When excluding implausibly fast responses as an exploratory analysis, age is associated with faster responses. According to the second hypothesis, participants who reacted more flexibly to negative feedback showed higher RL scores than participants who reacted less flexibly. Only partly in accordance with our expectation, this relation was most pronounced in adults. Third, we hypothesised to find an inverted-U relation between age and the number of flexible reactions, meaning that adolescents should react most sensitive to negative feedback. Contrary to our expectation, we found no general differences in flexibility between age groups. Nevertheless, as expected, adults reduced their responsiveness to negative instrumental feedback over the course of the experiment, indicating that they adapted their response strategy, unlike early-adolescents and mid-adolescents. Fourth, we found that working memory scores were associated with RL and flexible reactions, while NFC did not show any associations. Taken together, RL seems to improve during adolescence, especially between the ages of 10 and 14, and may be supported by proficient use of instrumental feedback. Below, these two aspects and their relation are discussed in more detail.

Our cross-sectional developmental results are in line with some previous research using different kinds of RL tasks. For instance, Crone et al.^25^ identified children between 8 and 11 years of age, compared to adolescents and adults, as the group performing worst in RL. In their task stimulus-outcome associations changed continuously after two to four correct trials. In a similar continuous task with reversals after six to ten trials executed by children between 8 and 12 years as well as adolescents between 13 and 16 years, children also showed lower performance than the adolescent group^29^. Although we used a blocked design, our finding that adolescents between 10 and 14 years show the highest performance increase may therefore extend to younger participants as well. Hauser and colleagues^34^, who did not include participants below 12 years, found that adolescents (12 to 16 years old) did not differ from adults in the number of switches in their response behaviour when contingencies reversed after at least six correct choices. Similarly, we also only observed small performance differences between older adolescents and adults. Van der Schaaf and colleagues^27^ tested RL performance in four age groups (10 to 11 years vs. 13 to 14 years, 16 to 17 years, and 20 to 25 years) with a task that changed associations after four to six correct responses. They also found the strongest increase in performance in 10- to 11-year-olds and decreasing improvements for 13- to 14-year-olds while performance was similar in the two oldest age-groups. Summing up, these studies support our finding that RL accuracy may reach a ceiling in late adolescence and that the highest gain in the ability to revise associations happens during early adolescence. This is in line with the observation that executive function more broadly improves more strongly in early adolescence compared to later adolescence^47^.

Complementing these observations, we explored in how far the development of RL in adolescents can be explained by pubertal development. We tested the influence of pubertal development with self-reported puberty scores, which are inherently more imprecise than external medical assessments^48^. Notwithstanding this caveat, we found that progress in pubertal development is related to higher RL performance amongst adolescents. These results may indicate that pubertal changes contribute to the observed improvements, but further studies are needed to examine the influence of puberty on learning measures more thoroughly.

Besides age or pubertal development, sex explains some differences in reversal learning performance in our study, with female participants performing better than male. There may be a genuine difference between female and male RL, or the higher performance of girls and women may point to the fact that females mature earlier than males in some regards, e.g., in grey matter development^49^ or structural connectivity^50^, and show earlier changes of reward-related behaviours^51^. This would further support the cross-sectional developmental differences observed in our study.

Furthermore, we found better RL performance to be related to higher working memory capacity irrespective of age, i.e., all participants similarly profited from higher executive function. Older participants had higher working memory scores, which could be related to the cross-sectional increase in RL performance we found. This is in line with research showing that the ability to retain and manipulate information in working memory promotes cognitive flexibility^52^. Interestingly, participants with higher working memory scores also tended to react more flexibly, pointing to the importance of cognitive capacity for flexible behavioural adaptation. In summary, previous behavioural research supports our result that flexibly relearning stimulus-outcome associations is most challenging for adolescents between 10 and 14 years. Still, the improvements in RL performance during early adolescence may be limited to certain tasks that are tailored in a specific way. On the one hand, they may not extend to less taxing tasks such as associative learning without reversals: For example, van den Bos et al.^21^ did not find differences in associative learning performance and response times between 8- to 11-, 13- to 16- and 18- to 22-year-olds. On the other hand, learning differences between older adolescents and adults may be more nuanced than apparent in a simple measure as the sum of correct responses, or require a higher degree of task difficulty. For instance, in the study by Palminteri and colleagues^26^ adolescents (12 - 17 years) and adults performed a RL task that either provided rewarding or punishing feedback and additional counterfactual information or not. Adolescents performed only worse when counterfactual information was provided that adults could make use of, while adolescents seemingly did not take this information into account. Furthermore, adolescents learned less than adults from punishing feedback, while there was no difference in the rewarding condition. Thus, there seem to be more nuanced differences between mid-adolescents and adults not reflected in overall RL performance. Responding to negative feedback is one such possible nuance that we investigated in our study and discuss in the next section.

To better understand the development of RL, we examined its relation to the tendency to react flexibly to negative instrumental feedback, i.e., to change one’s answer after receiving the feedback that the former response to a stimulus was wrong. Our study points to a supporting role of flexible reactions for RL for all participants that might change its influence during development. We expected that mid-adolescents should exhibit most flexible reactions although they might not necessarily profit from these responses in terms of better RL. Against this expectation, the number of flexible reactions did not change with age neither in terms of an inverted U-shaped or linear relation. Complementing this result, although there was no linear increase in flexible responses across the complete age range, in an exploratory analysis we found a significant increase of flexible responses between 10 and 14 years and 18 and 22 years. The increase in early adolescence is in line with the proposal of DePasque and Galván^18^ that during puberty the prefrontal control is developed that allows to process negative feedback more efficiently.

We based our hypothesis on the study by Van der Schaaf and colleagues^27^ which showed an inverted-U relation between age and reactions to negative instrumental feedback - a measure close to our flexible reaction score. Similarly, Hauser et al.^34^ found that adolescents learned more quickly from negative instrumental feedback than adults. The study by Waltmann et al.^28^ included participants from 12 to 45 years and also showed that younger participants changed their responses after negative feedback more readily than older participants. However, these studies show significant differences to ours. In Hauser and colleagues’ study^34^, the underage sample was on average 14.7 (*SD* = 1.3, *range* = [12, 16]) years old, lacking the younger adolescents we included. Van der Schaaf et al.^27^ found that especially adolescents between 16 and 17 years learn most strongly from negative instrumental feedback, compared to both, children and adults. In contrast to our study, participants were not directly informed of the correct response but rather had to deduce it by comparing their predicted outcome with the actual outcome. It could therefore be that adolescents are better than adults and children in their ability to react flexibly to counterfactual feedback but not so for transparently displayed instrumental feedback. This is partially supported by the models of reasoning Palminteri et al.^26^ used to explain their previously mentioned results, since they found that adolescents may not profit as much from counterfactual information as adults. On the other hand, in the study by Waltmann and colleagues^28^ direct instrumental feedback was provided just as in our paradigm. Nevertheless, their task was designed in such a way that more flexible responses to negative feedback meant not being sensitive enough to former positive feedback, i.e., being overall less reinforcement sensitive, corresponding with worse learning performance in the stable phases between reversals. In our study, adults may have reacted as flexible as adolescents because we looked at switching behaviour at the beginning of the learning blocks, so that changing responses after negative feedback was adaptive. Accordingly, flexible reactions were related to better RL performance irrespective of age. Examining the age groups separately, we found that this relationship was most prominent in adults, hinting at their ability to make use of flexible responses most efficiently.

This assumption is further supported when considering the rate of flexible reactions across the three blocks. Only adults showed an adaptation of their response strategy to the task demands, reflected as a decrease in flexible reactions over the course of the experiment, while such changes were less clear or absent in early- and mid-adolescents, respectively. This decrease in flexibility among adults is not related to a decline in overall performance but rather suggests they use the full extent of the provided information, including shifts in the optimal strategy, more effectively than adolescents. In the paradigm, adapting responses immediately to negative feedback is generally successful, but this strategy can be improved upon by recognizing that some feedback at the beginning and end of a learning block is invalid and should not be followed. Adults reacted more flexible overall but may have intentionally reduced their flexible reactions toward the end of the paradigm, having realized the occasional invalidity of feedback. This represents a higher-order strategy requiring the ability to judge the validity of feedback before adapting responses, which we expected only adults to achieve. Thus, adults may use complex, less immediate or tangible sources of information better than adolescents- in the case of our study in form of slow changes in optimal strategy, or as previously reported in form of counterfactual feedback^26^. Accordingly, a review of computational modelling studies suggests that the adaptation of learning rates might increase with age, i.e., adults are better in optimizing how heavily their responses are influenced by recent feedback^24^. Studies using other measures of learning and adaptation also showed that only older adolescents and adults can properly adjust their learning strategy to specific environments, e.g., in a recognition memory task^53^, during decision making^54,55^ and in an estimation and choice task^56^. As Nussenbaum and Hartley^24^ point out, the adult ability to use the most effective response strategy may explain why on the surface, instrumental learning studies are inconsistent regarding the extent to which feedback is used by different age groups. Task specifics may determine if it is better to include recent negative or positive feedback, or not, thus influencing if adolescents or adults react more flexibly. Therefore, in the last paragraph, we will discuss the strengths and limitations of our paradigm to make some suggestions which aspects might be considered in further studies.

In designing this paradigm, we tried to find a balance between task difficulty, so that developmental differences can be measured, and feasibility, so that participant drop-out is not too high. The results indicate that we achieved this goal since we found age-dependent differences and retained 73% of the sample. Nonetheless, the paradigm could be adapted to increase the number of younger participants who manage to learn the initial associations. Moreover, we did not manipulate the learning context in a way that allowed to investigate its influence on RL. Besides the role of instrumental feedback, we initially planned to investigate if adults or adolescents are more open to contextual clues that inform about contingency changes. After beginning the data collection, we realised that the design only allows to test the relation of contextual information on probabilistic learning, but not on RL, since the intended variables are confounded. Future replications or adaptations of this paradigm may improve on this.

Furthermore, there are some caveats to consider when interpreting our results. First, since the study was run online, some variance in the participants’ performance might be attributed to their different environments at home, the devices they used and reliability of their internet connection. When excluding implausibly fast responses, the results regarding response times change, indicating that quality control is necessary for such online experiments. If younger adolescents had more difficulties with using their computers than the university students, that mostly make up the adult participants, this might have confounded the results. We tried to avoid this by calling the participants via phone, providing instructions orally and giving enough time to ask questions regarding the task or procedure. Furthermore, we made sure that the experiment ran properly on the participant’s web browser, asking the parents’ help, if necessary. Although we do not believe that there are other factors that may have systematically influenced the data, ideally, the results should be replicated in a more controlled environment, such as a laboratory. Second, the paradigm should be repeated in a longitudinal study, to complement our cross-sectional results.

A strength of our paradigm is that we measured the role of responses to negative instrumental feedback separately from RL. This way, we were able to observe that the ability to respond flexibly seems to increase between 10 and 14 years and is related to better RL performance in all participants, but most prominently in adults. Nevertheless, the paradigm’s design determines the adaptive value of processing negative feedback, and therefore may influence the age-related results. As discussed above, inconsistent findings related to adolescent vs. adult advantages during RL may be the result of differences in the consequences of flexible responses to negative feedback. If changing responses is adaptive and reflects in better task performance, like in our paradigm, adults may show more flexible responses due to their ability to optimize behaviour in varying environments. If, however, flexible responses are in competition with more adaptive behaviour, adolescents may be most flexible because of a higher openness to new information or a higher expectation of environment volatility^56^, depending also on the type and value of feedback^27,29,57,58^. Therefore, on the one hand, the advantage or disadvantage of adolescents compared to adults in RL might depend substantially on task properties, while on the other hand adolescents might gain substantial improvements in cognitive flexibility compared to children. Keeping this in mind, future paradigms might try to manipulate the adaptivity of flexible behaviour within a paradigm and consider its possible non-linear effects on RL in different age groups.

Moreover, recommendations for educational settings can be deduced from this study. Younger adolescents particularly struggled to discern valid from invalid feedback, which hindered their ability to learn stimulus-outcome associations. Clear, unambiguous feedback may be crucial for their learning, while training the ability to identify patterns in noisy information could be a valuable educational focus for this age group. Furthermore, the difficulty younger participants had in learning associations, compared to the stronger performance of older participants, highlights the importance of calibrating task demands to children’s and adolescents’ developmental capacities in both research and educational contexts. It was shown that game-based flexibility training for children improved untrained executive functions which even transferred to better sentence comprehension^59^. In sum, the study showed that learning and its components develop in a complex pattern, requiring age-appropriate learning situations for individuals to excel.

## Methods

### Participants

Seventy-four male and 138 female participants aged 10 to 22 participated. They were recruited via social media, the city’s birth-registry, former participants’ databank, and word-of-mouth. Exclusion criteria were no internet access, uncorrected refractive errors, deuteranopia or protanopia (“red-green deficiency”), diagnosed special educational needs or insufficient German proficiency. Participation was compensated with a 10€ voucher. Informed consent was obtained online from adults or legal guardians. The study was approved by the ethics committee of the Technical University Dortmund (date of approval 15.03.2021) and is in accordance with the declaration of Helsinki. To test hypothesised differences between age groups, we categorise early-adolescents as between 10 to 14 years, mid-adolescents between 14 and 18 years and adults as above 18 in all analyses. This separation is based on the studies reviewed in the introduction and evenly splits the age range in the sample.

Of the 212 participants, data from 13 participants could not be used due to errors: Data of four participants was excluded because they did not respond for more than 10 consecutive trials. Three participants terminated the experiment prematurely. Data from one participant could not be used due to technical issues. One participant was excluded due to giving false information. Further four participants were excluded because of missing data.

### Reversal Learning Paradigm

The RL paradigm was developed using the PsychoPy Builder (Version 2020.1.2^60^) and was run on the repository and experimental platform Pavlovia^61^, accessed through the participants’ web browser. In the experiment, participants learned to predict whether sweets, which could be bought in kiosk A or B, cause a stomach-ache or not in a fictitious group of friends based on probabilistic feedback. A trial was set up as follows: After a fixation cross, participants saw sweets framed by the picture of either kiosk A or B for one second. Then, participants were prompted to predict whether the sweets cause stomach-ache (“Yes”) or not (“No”) within three seconds. After a delay of half a second, feedback was presented for three seconds. The feedback was both instrumental (a green frame marking their choice if they are correct, a red frame if not) and valence-based (written feedback that their friends do or do not get a stomach-ache accompanied by a corresponding happy or sad emoji).

**Fig. 7:**
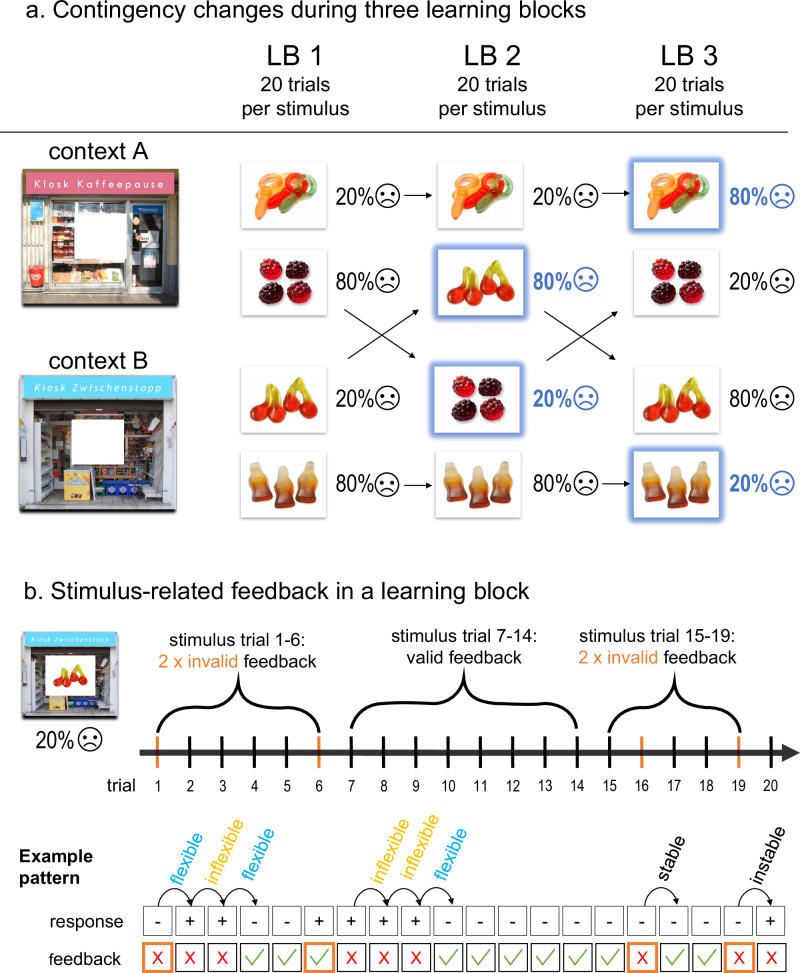
Trials In The Three Learning Blocks. The figure shows how stimuli changed their associated outcome throughout the paradigm. (**a**) Blue stimuli changed their associated outcome compared to the previous learning block. The percentage next to the stimuli indicates how often a stimulus was associated with stomach-ache in a particular block. The vertical or diagonal arrows show whether a stimulus remained in the same context or changed it between learning blocks. (**b**) Within one learning block, there were three phases depending on the occurrence of invalid feedback (yellow), exemplified for a single stimulus. A response change after negative feedback within the first ten stimulus presentations was counted as flexible reaction. An example pattern of fictive participant responses and resulting feedback demonstrates the measurement of flexible reactions: In trial 1, the participant correctly responds (−) but gets invalid negative feedback. He changes his response to (+) in trial 2, thereby reacting flexibly to the negative feedback (although this answer is false). After valid negative feedback, he sticks with his response (+) in trial 3, thus not reacting flexibly. Repeated negative feedback prompts him to change the response back to (−) in trial 4, which counts as flexible response. He gets valid positive feedback and correctly answers (−) again in trial 5. Despite valid positive feedback, he erroneously changes his response to (+) in trial 6. He receives invalid positive feedback and sticks with the response (+) for three trials, despite valid negative feedback in trials 7 and 8. Only after the negative feedback in trial 9, he changes his response in trial 10, reacting flexibly again. In sum, this participant responded flexibly three times, while negative feedback was given six times, so that his quota of flexible responses is 3/6 = 0.5.

Participants completed three learning blocks (LB 1–3), each consisting of 80 trials. During each block, they learned the outcomes of four types of sweets: two presented in kiosk A and two in kiosk B. The assignment of sweets to contexts and outcomes was randomized per participant. As shown in Fig. 7, each sweet caused stomach-ache in either 20% or 80% of its 20 presentations per block, balanced across contexts. This resulted in four invalid feedback trials per sweet per block.

To manipulate the feedback’s informational value, two invalid feedback trials were included in the first six presentations of each sweet. Invalid feedback could either be negative (incorrectly marking a correct response as wrong; Fig. 7b, trial 1) or positive (incorrectly marking an incorrect response as correct; Fig. 7b, trial 6). With this design we assessed how flexibly participants adjusted their responses after feedback (see analysis plan for details on the outcome measure “flexible reactions”). The remaining two invalid feedback trials occurred toward the end of the block (during trials 15–19) to evaluate how well participants retained learned associations when faced with invalid feedback. In the middle trials (7–14), only valid feedback was provided, allowing participants to learn the predominant stimulus contingencies. This feedback pattern applied to all four sweets and across all three learning blocks.

Stimulus presentations were pseudo-randomized to prevent the same stimulus from appearing in consecutive trials. To test adaptation to changes in stimulus-outcome associations, the same stimuli were used across blocks, but their outcome associations changed without prior notice. As shown in Fig. 7, two stimuli remained in the same context but changed outcomes between LB2 and LB3. The other two switched contexts in each block and changed outcomes between LB1 and LB2. Context switches were introduced to explore their impact on probabilistic learning, which will be addressed in a future publication.

In addition, there were brief test blocks after LB1 and LB2 during which the participants had to indicate which stimulus outcome they expect without receiving feedback. In the first test block (TB1), the four stimuli were presented in their initial learning context (i.e., the kiosk they were presented in during LB1) five times each to test whether the associations were learned properly. During the second test block (TB2) all stimuli were presented in all contexts in order to assess whether the reversed stimulus-outcome associations in LB2 were recalled.

In order to ensure that participants properly learned the stimulus-outcome associations in the first learning block, we defined an initial learning threshold they had to reach for the paradigm to continue. The learning performance was considered sub-threshold if the participants were correct in less than 21 out of the 32 possible correct answers in the middle eight trials of stimulus presentations’ during the first learning block (i.e., the probability that they answered randomly is less than 5% according to binomial distribution).

### Questionnaires

Before scheduling the experimental session, participants, and an accompanying adult in case of minors, answered several questionnaires. These included demographic questions, the short version of the depression anxiety stress scale (DASS-21)^62^, 10-item short version of the Big Five inventory^63^, need for cognition scale^44^, emotion regulation questionnaire^64^ and the behavioural inhibition system/ behavioural approach system questionnaire^65^.

In addition, underage participants answered the strengths and difficulties questionnaire^66^ and the pubertal development scale^67^. The NFC and pubertal development scale are used in the analysis here. The remaining questionnaires may be analysed at a later point. The pubertal development scale consists of four different questions for male or female participants that ask for a self-report on characteristic bodily changes occurring during puberty. The average score from the answers is calculated for use in the statistical analysis.

### Working Memory Assessment

Participants’ working memory was assessed with a digit span and backward digit span test, adapted from the Wechsler intelligence scale^68^. The experimenter read number sequences to the participant over the phone. The participant had to retain the sequence in memory and recite it immediately after. They were explicitly asked not to note the sequences down. Sequence length increased every second time and ranged from two to nine digits for the forward and two to eight digits for the backward version. The number of correctly recited sequences is averaged for both versions.

### Procedure

Questionnaires and the consent form were provided via Qualtrics (Qualtrics, Provo, UT, USA). The experiment was remotely administered via telephone and web browser. Before attending the experiment, participants and their parents (in case of minors) independently answered the questionnaires. Immediately before the experimental paradigm started, they underwent the working memory assessment and reported what web browser they used. To make the environment more comparable, all participants were asked to turn of disturbing devices and sit in a quiet atmosphere, if possible. Researchers followed a script for the instruction of participants and tested whether they understood the study task by asking them to respond to an example trial. Participants received their renumeration after completing the experiment. Several experimenters administered the study after being trained sufficiently. The study took approximately 35 minutes, of which 20 minutes were spent on the RL paradigm.

### Statistical Analysis Plan

In this manuscript we focus on preregistered hypotheses on RL (research question b and parts of c and f in the preregistration, https://osf.io/ep5ua). Some of the respective analyses are reported in the supplementary material for completeness.

We calculated the variables used in the statistical analysis in the following way. Reversal learning performance was calculated for stimuli that changed the stimulus-outcome association compared to the previous learning block. We used the first 14 trials with these reversed stimuli per block to calculate a quota between 0 and 1 for how well participants learned the reversed stimulus-outcome associations. The later six trials did not factor into this calculation since they were intended to test the stability of participants’ responses specifically. Flexible reactions are also calculated as a fraction between 0 and 1. A reaction is counted as flexible if a participant changes the response to a certain stimulus in the next trial after receiving negative feedback within the first ten stimulus presentations within the learning block. We include the ten nine trials since the effects of invalid feedback may extend beyond the trial right after (see for example Fig. 7. B trial 6 – 9). The quota of flexible reactions is then calculated as the number of flexible answers divided by the number of all trials in which negative feedback was given within the first ten stimulus presentations and is averaged for all stimuli of a learning block. That is, participants who never change their answer after negative feedback would get a score of 0, while participants who always change their response after negative feedback would receive a score of 1. Note that a response counts as flexible if the participant changes his response after negative feedback, irrespective of the correctness of the answer. Therefore, flexible reactions measure the openness to change behaviour according to negative feedback, independent of reversal learning performance. Furthermore, erroneous changes in responses, without prior negative feedback, are not counted towards the flexible reactions. Therefore, the measure of flexible behaviour is discernible from impulsive or erroneous changes in responses. As planned in the preregistration, to further assure the independence of variables in the regression models, the measures of the quota of flexible responses are adjusted to the respective outcome variables. The RL performance of the two stimuli that changed their contingency in LB2 and LB3, respectively, are confounded with the quota of flexible reactions in reaction to these stimuli. Hence, the relation of flexible reactions with RL performance is tested by only including the quota of flexible reactions for all stimuli in LB1 and the stimuli in LB2 and LB3 that did not change their outcome-association. When analysing flexible reactions as a dependent variable, the values from all LBs and stimuli are used.

Response times were calculated as the median of reaction times of trials with a correct response for all four stimuli in one learning block. Response times were not normally distributed in 96% of participants according to an omnibus test of normality^69^. Hence, the logarithm of the response times was used in the analysis. Since the web browser used to run the experiment may influence the accuracy of response time measurement^70^, we added the type of browser as a factor in analyses of reaction times.

For hypotheses proposing a linear relation between independent and dependent variable, linear regression models are fitted to the data of those participants that reached the initial learning threshold. If the hypothesis stipulated an inverted-U relation, quadratic regression terms are used. As the sex distribution in the sample was not equal, we added *sex* as a covariate in additional analyses to exclude that results are driven by sex effects. We consider a test to be significant if it is expected to reach a false positive rate of 5% or below in the long run.

As an exploratory analysis in addition to linear regression, we also fitted linear basis-spline regression models for developmental research questions. This allows for more flexibility in investigating the relation between age and the outcome variable. On the one hand, this method allows for a more flexible fit than a standard regression and may better account for non-linear developmental effects. On the other hand, the shape of the regression line is more constrained than when fitting a higher-order polynomial regression. Two knots at 14 and 18 years of age were chosen so that the resulting slopes represent the development in pre- and early puberty, during mid-puberty, and during young adulthood. Furthermore, we used the pubertal development scores as an alternative predictor to age and refitted models with this predictor instead because the relation between age and RL may be further understood by taking into account the pubertal development in underage participants. Puberty does not progress with the same continuity as age and reaches a natural end point, therefore self-reported puberty and RL may be related in a different way than age and RL.

## Supplementary information

Supplemental Material

## Data Availability

The data that support the findings of this study are available from the corresponding author, CB, upon reasonable request.

## Code availability

The code used to analyse the data are publicly available from the OSF project accompanying this publication: https://osf.io/8mc5r/files/osfstorage.
