# Supplementary material for: Reversal learning is influenced by cognitive flexibility and develops throughout early adolescence
Source: NPJ Sci Learn. 2025 May 12;10:27. doi: 10.1038/s41539-025-00308-3 (PMC12069702; doi:10.1038/s41539-025-00308-3)
Supplement: Supplementary file 1 — Supplemental Material [file 41539_2025_308_MOESM1_ESM.pdf]

Supplementary Material

Supplementary Table 1.

Number Of Excluded Subjects With Low Performance

Age	10	11	12	13	14	15	16	17	18	19	20	21	22	Total
Number of excluded participants	9	6	2	5	6	4	0	4	4	1	6	2	5	54

Supplementary Table 2.

Performance in learning blocks, split by age groups

Age group	LB	Coefficient	SE	95% CI	p
Early adolescent	LB 1	0.70	0.02	[0.66, 0.73]	< .001
	LB 2	-0.08	0.02	[-0.12, -0.03]	.002
	LB 3	-0.11	0.02	[-0.16, -0.06]	< .001
Mid adolescent	LB 1	0.70	0.02	[0.66, 0.73]	< .001
	LB 2	0.005	0.03	[-0.05, 0.06]	.858
	LB 3	-0.05	0.03	[-0.11, -0.003]	.037
Adult	LB 1	0.71	0.01	[0.68, 0.73]	< .001
	LB 2	0.01	0.01	[-0.02, 0.04]	.484
	LB 3	-0.02	0.02	[-0.05, 0.01]	.263

Supplementary Figure 1.

Violin Plot For Performance In The Three Learning Blocks, Split By Age Group

Learning block one to three are plotted on the x-axis for early-adolescents, mid-adolescents and adults. Performance in learning stimulus-outcome associations in the respective learning block is plotted on the y-axis. Horizontal bars are means, the density is given by the shape of the violin plot and data points are slightly jittered.

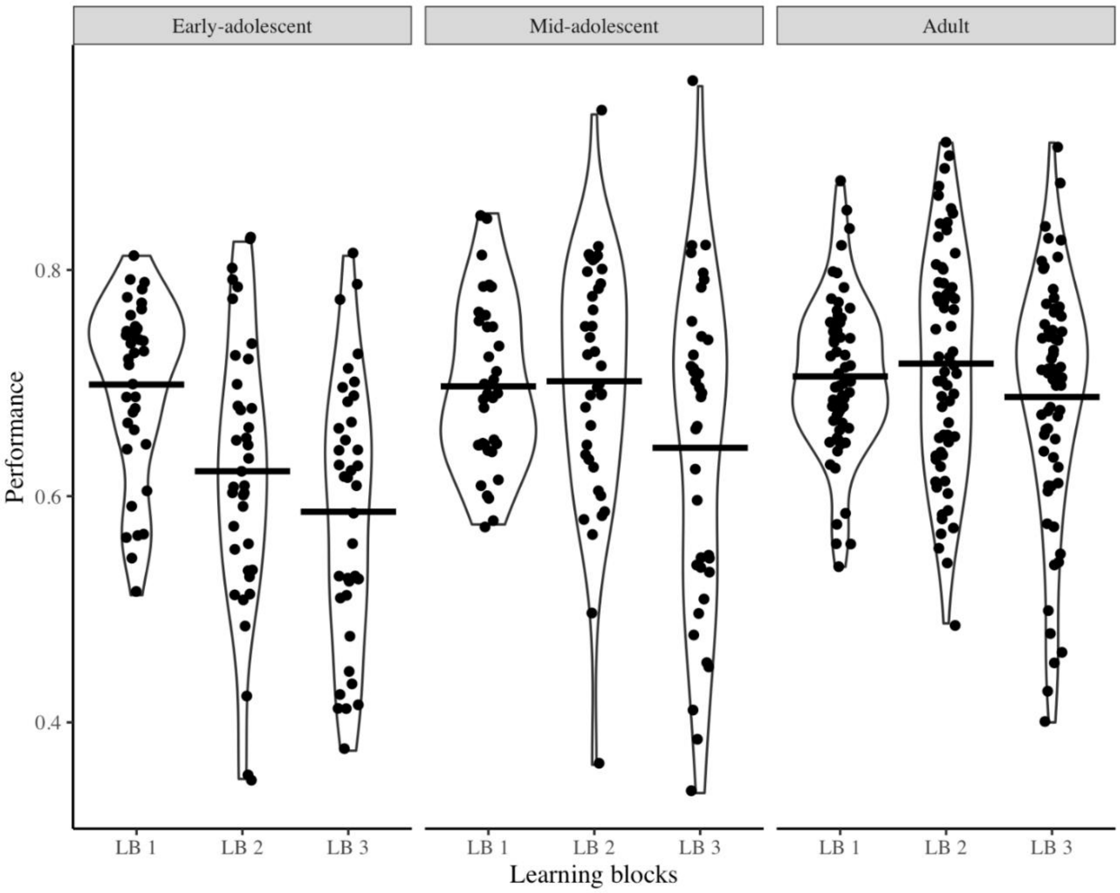

Basis Spline Regression Model for Age and RL Results

Supplementary Table 3.

Coefficients from basis spline regression

Basis function	Coefficient	SE	95% CI	P
Intercept	0.46	0.04	[0.37. 0.54]	< .001
10 to 14	0.19	0.06	[0.07, 0.31]	< .001
14 to 18	0.18	0.05	[0.09, 0.28]	< .001
18 to 22	0.24	0.05	[0.14, 0.34]	< .001

Basis Spline Regression Model for Age and Flexible Reactions Results

Supplementary Table 4.

Coefficients from basis spline regression

Basis function	Coefficient	SE	95% CI	P
Intercept	0.50	0.03	[0.44, 0.57]	< .001
10 to 14	0.10	0.05	[0.01, 0.19]	.028
14 to 18	0.06	0.04	[-0.01, 0.14]	.079

18 to 22	0.10	0.04	[0.02, 0.18]	.012

Main Results after Removing Outliers

We report the main results after removing data points with a Cook's distance higher than three times the mean distance for the initial sample. The coefficient for age on RL performance is 0.01 (*SE* = 0.003, *95%CI* = [0.01, 0.02], $p < 0.001$; 3 outliers removed). The coefficient of age in the linear regression for log-transformed response times and the used web browser as additional variable is -0.01 (*SE* = 0.01, *95%CI* = [-0.03, 0.004], $p = 0.16$; 13 outliers removed). The coefficient for flexible reactions' effect on RL is 0.43 (*SE* = 0.08, *95%CI* = [0.27, 0.6], $p < 0.001$; 9 outliers removed). The coefficient of age, as a linear term, for the model of flexible reactions is 0.01 (*SE* = 0.01, *95%CI* = [-0.01, 0.02], $p = 0.21$; 10 outliers removed) and as a quadratic term 0.001 (*SE* =0.01, *95%CI* = [-0.02, 0.02], $p = 0.93$).

Gender Differences

Using a Fisher's Exact test for the share of female/male participants per age group, we did not find significant differences in how the gender is distributed for different ages ($p = .331$).

Supplementary Table 5.

Gender Distribution By Age

Age	10	11	12	13	14	15	16	17	18	19	20	21	22	Total
N female	3	8	5	9	5	6	8	7	3	10	10	16	7	97
N male	1	5	3	3	3	3	4	3	5	9	2	6	7	45

In what follows, we report several analyses with gender as a covariate. Table A3 shows the results of adding gender to the model for the dependent variable RL and the independent variable flexible reactions. Table A4 shows the model of flexible reactions with the predictors age, squared age and gender.

Supplementary Table 6.
Linear Regression For RL With Flexible Reactions And Gender

Term	coefficient	SE	95% CI	p

Intercept	0.40	0.05	[0.29, 0.51]	< .001
Flexible reactions	0.36	0.09	[0.18, 0.53]	< .001
Gender	0.05	0.02	[0.00, 0.09]	.09

Supplementary Table 7.
Linear Regression For Flexible Reactions With Age And Gender

Term	coefficient	SE	95% CI	p
Intercept	0.40	0.19	[0.01, 0.78]	.044
Age	0.02	0.02	[-0.03, 0.06]	.466
Age^2	0.00	0.00	[0.00, 0.00]	.557
Gender	0.02	0.02	[-0.02, 0.05]	.305

Reversal Recall

We tested whether the ability to recall reversals in stimulus-outcome associations right after they changed is predicted by the participants' age. There does not seem to be a significant association between age and reversal recall (coefficient = -0.002, SE = 0.006, 95% CI = [-0.01, 0.01], p = 0.7).

Valence and Reversal Learning

We tested the effect of valence (negative/ stomachache or positive/ no stomachache) on RL performance in separate linear regressions for early-adolescents, mid-adolescents, and adults. There is no significant relation in early-adolescents (*coeff* = -0.016, *SE* = 0.032, *95%CI* = [-0.08, 0.05], *p* = 0.62), mid-adolescents (*coeff* = -0.02, *SE* = 0.03, *95%CI* = -0.08, 0.04], *p* = 0.51) and adults (*coeff* = -0.04, *SE* = 0.02, *95%CI* = [-0.08, 0.004], *p* = 0.07).

Associations with Questionnaire Data

We tested whether the RL performance or flexible reactions are associated with psychological traits measured in the DASS and BIS/BAS questionnaires. Correlations are shown in Table S5.

Supplementary Table 8.

Correlation Table For RL And Questionnaires

	RL	flexible reactions	DASS stress	DASS anxiety	DASS depression	BIS	BAS drive	BAS fun seeking	BAS reward responsiveness	NFC
RL	1									
flexible reactions	0.45*	1								
DASS stress	-0.01	-0.1	1							
DASS anxiety	0.05	-0.06	0.56	1						
DASS depression	-0.03	-0.01	0.56	0.56	1					
BIS	-0.04	-0.07	-0.35	-0.27	-0.21	1				
BAS drive	-0.02	-0.05	0.06	0.15	0.25	-0.01	1			
BAS fun seeking	0.01	0.13	0.03	0.05	0.11	-0.18	0.21	1		
BAS reward responsiveness	0.01	-0.13	-0.06	0.11	0.16	0.16	0.46	0.21	1	
NFC	0.12	0.12	-0.08	-0.06	-0.2	-0.001	-0.33	-0.05	-0.26	1

Note: There are no significant correlations between RL and the questionnaire variables or flexible reactions and the questionnaire variables.
